# Hemorrhage From a Pituitary Macroadenoma After a Minor Trauma

**DOI:** 10.4021/jocmr2009.11.1272

**Published:** 2009-12-28

**Authors:** Mohammad Sami Walid

**Affiliations:** a Medical Center of Central Georgia, 840 Pine Street, Suite 880, Macon, GA 31201, USA. Email: mswalid@yahoo.com

## To the Editor

Pituitary hemorrhage is a rare and possibly life-threatening condition caused by acute bleeding in the pituitary gland, usually within a macroadenoma. In this letter we report a case of a large pituitary hemorrhage that fared well with relatively mild sequelae.

**Figure 1:**
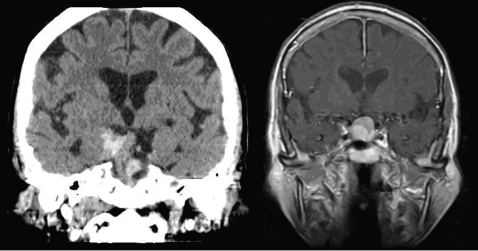
Coronal views of head CT (left) and MRI T1 with contrast (right) showing hemorrhage in the pituitary macroadenoma.

**Figure 2:**
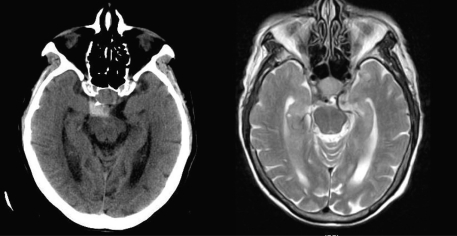
Axial views of head CT (left) and MRI T2 (right) showing the pituitary hemorrhage (CT) and the pituitary macroadenoma (MRI).

**Figure 3:**
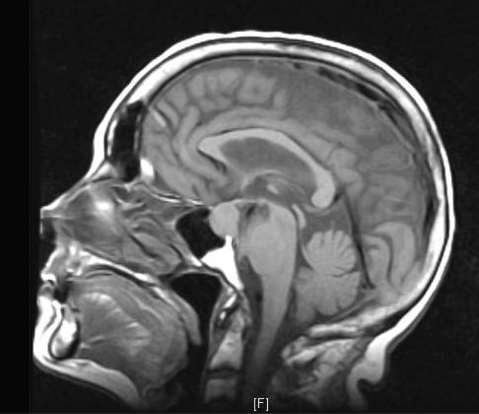
Sagittal view of head MRI T1 showing the pituitary macroadenoma.

An 80-year-old Caucasian woman presented with severe headaches of two days duration that started after a fall in the yard without loss of consciousness. She took aspirin for the headaches without effect. CT of the head showed a suprasellar hemorrhage. MRI showed pituitary enlargement, 20 x 19 x 16 mm, with enhancement in the caudal right aspect of the pituitary gland (Fig. 1-3). The pituitary gland extended cephald to about the optic chiasm. There was blood within the right suprasellar cistern extending into the prepontine cistern along the clivus. On physical exam there were no remarkable findings except for a blood pressure of 148/105 mmHg. Her white blood cell count was 6000 /mm^3^, hemoglobin 13 g/dl, hematocrit 39%, platelets 214000 /mm^3^, prothrombine time 15.7 seconds and INR 1.22. The patient had no significant past medical history. She reported no breast discharge, vision problems or seizures. The patient did not smoke or drink and was on no medications and looked younger than her age. The possibility of a ruptured aneurysm was investigated and excluded on a four-vessel cerebral arteriogram. The clinical and radiological picture was more consistent with a bleeding from a macroadenoma than with a pituitary apoplexy because of the subtle changes in the hormonal homeostasis. The patient was first admitted to ICU then transferred to the floor after four days and discharged two days later. Four years afterward the patient was seen on a follow-up visit; she was on levothryoxine, bromocriptine and risedronate and was doing fine.

This case is interesting because of its presentation. Few similar cases of pituitary hemorrhage or apoplexy after a minor trauma were reported in the literature [1-3]. An adenoma may grow silently in a patient and reach considerable measurements and then suddenly reveal itself after a minor trauma in form of hemorrhage or apoplexy. In our case, what may have contributed to the radiological picture is that the patient with evident brain atrophy (as seen on CT) tried to treat pain with aspirin, a strong antiaggregant. This probably prevented a small vascular injury in the pituitary adenoma from occluding itself turning a microscopic-size bleeding into a radiologically-sizable one. In a recently reported study by Kim et al, out of thirty two pituitary bleeding cases, three patients had a head trauma history as a result of traffic accidents within the previous three weeks and two patients had aspirin [4].

The pituitary gland is a small but vital organ and should be suspected in every patient presenting with abrupt severe headaches with or without ophthalmological symptoms. Radiological investigation of such cases, especially elderly patients, is imperative even after a minor traumatic trigger.
